# The use of tissue sealant in reducing urethrocutaneous fistula event following hypospadias repair: A systematic review and meta-analysis

**DOI:** 10.1016/j.amsu.2022.103707

**Published:** 2022-05-13

**Authors:** Tomy Nurtamin, Johan Renaldo, Yudhistira Pradnyan Kloping, Ilham Akbar Rahman, Lukman Hakim

**Affiliations:** a Department of Urology, Faculty of Medicine, Universitas Airlangga, Dr. Soetomo General-Academic Hospital, Mayjen Prof. Dr. Moestopo No.6-8, Surabaya, 60286, Indonesia; b Dr. Soetomo General-Academic Hospital, Mayjen Prof. Dr. Moestopo No.6-8, Surabaya, 60286, Indonesia; c Department of Physiology, Faculty of Medicine, Universitas Halu Oleo, Kampus Hijau Bumi Tridarma Anduonohu, Kendari, 93232, Indonesia; d Universitas Airlangga Teaching Hospital, Dharmahusada Permai, Mulyorejo, Mulyorejo, Surabaya, 60115, Indonesia

**Keywords:** Hypospadias repair, Tissue sealant, Urethrocutaneous fistula

## Abstract

**Background:**

One of the most frequent complications following hypospadias repair is urethrocutaneous fistula (UCF) event. Tissue sealant has been introduced as a means to reduce UCF. However, reports regarding its benefits are varied. Thus, we initiated a systematic review and meta-analysis to investigate its role in reducing UCF following hypospadias repair.

**Methods:**

We completed a systematic search through the Embase, MEDLINE, and Scopus databases for studies assessing postoperative complications in hypospadias patients undergoing urethroplasty with and without tissue sealant. Cochrane risk of bias 2 (RoB 2) tool was used to assess the quality of randomized clinical trials (RCTs), while the observational studies were assessed with Newcastle-Ottawa Scale. The primary outcome analyzed in this study was UCF, while secondary outcomes consisted of postoperative complications such as edema, infection, and wound dehiscence that increases the risk of UCF formation, measured using odds ratio (OR) with a 95% confidence interval (CI).

**Results:**

Six eligible studies comprising three RCTs and three non-randomized studies were included. Patients undergoing hypospadias repair with tissue sealant had lower UCF events (OR = 3.27; 95% CI 1.92–5.58; p < 0.0001). Likewise other post-operative complications, tissue sealant group had a lower rate of edema (OR = 2.29; 95% CI 1.38–3.78; p = 0.001) and infection (OR = 3.87; 95% CI 1.55–9.70; p = 0.004). The difference in wound dehiscence was insignificant between the groups (OR = 2.08; 95% CI 0.21–20.55; p = 0.53).

**Conclusion:**

Tissue sealant can reduce UCF events following hypospadias repair as well as edema and infection that increases the risk of UCF formation.

## Highlights

• Tissue sealant can reduce UCF events following hypospadias repair.
• Edema and infection increase the risk of UCF formation.
• Tissue sealant provides a watertight surgical anastomosis right after application.
• Tissue sealant can resist hydrostatic pressure of urine at the surgical suture line.

## 1 Introduction

Hypospadias is a common congenital abnormality in the urethra, where the urethra meatus is located on the penis ventral side. The location of the urethra meatus could be in the distal, midshaft, and proximal of the penis [1,2]. Hypospadias occurs in about 1: 200 to 1: 300 male newborns [[3], [4], [5]]. The standard of care for hypospadias is surgical repair of the anatomical defect. Hypospadias repair is aimed to improve the cosmetic and functional aspects of the penis. Currently, several surgical techniques have been introduced. However, none of these techniques have been considered the gold standard [5,6]. The recommended surgical technique depends on the location of the urethral opening, the complexity of repair, technical modifications, and surgeon experience [[5], [6], [7], [8], [9], [10]]. Therefore, various outcomes and complications may occur following hypospadias repair [3,[9], [10], [11], [12], [13], [14]].

One of the most frequent complications following hypospadias repair is urethrocutaneous fistula (UCF). This complication remains a frustrating problem and challenging issue [3,[9], [10], [11], [12], [13], [14]]. The exact etiology of UCF remains less known. Localized infection and ischemia, edema, wound dehiscence, insufficient procedure, and improper tissue handling are thought to contribute to this complication [5,15,16]. Many techniques have been used to reduce UCF events and other associated complications that increase the risk of UCF formation following hypospadias repair [6,10]. One of the technique modifications carried out is the administration of additional materials during urethroplasty. Tissue sealant is a topical agent used as hemostasis and tissue adhesives [3,[11], [12], [13], [14], [15]]. It has been used and investigated to minimize complications following hypospadias repair. Numerous studies have demonstrated that tissue sealant may promote wound healing by filling gaps on the wound edges, reinforcing suture, reducing local infection, reducing inflammation, and promoting fibroblast proliferation following hypospadias repair [3,11]. However, the use of tissue sealant to reduce the UCF event following hypospadias repair has conflicting results [3,[11], [12], [13], [14], [15]]. As a result, we initiated a systematic review and meta-analysis to investigate the role of tissue sealant in reducing UCF following hypospadias repair.

## 2 Methods

### 2.1 Protocol and registration of the study

This study followed the guidelines of the Preferred Reporting Items for Systematic Review and Meta-Analysis (PRISMA) 2020 flow chart [16]. This study protocol was recorded in the PROSPERO international prospective register of systematic reviews (CRD42020198066) and Research Registry (reviewregistry1348).

### 2.2 Literature search and study selection

A systematic search was conducted in several databases through the MEDLINE, Scopus, and Embase databases up to February 2021. Relevant studies were searched using a Boolean operator with the following search strategy: (Tissue Sealant OR Sealant OR Tissue adhesive OR Fibrin Glue) AND (Hypospadias) OR (Urethroplasty).

### 2.3 Eligibility criteria

The search of this study was prioritized for individual studies that assessed the comparison of using tissue sealant, and without using tissue sealant in hypospadias patients undergoing urethroplasty. If a study met the following inclusion criteria, it was considered eligible: (1) English articles; (2) Randomized Controlled Trial (RCT), cohort, and case-control study design; (3) the data comparing postoperative complications between using tissue sealant, and without using tissue sealant were available. We excluded the studies of reviews, commentaries, letters, experimental animal studies, abstracts, and single-arm case series.

### 2.4 Data extraction and quality assessment

Three reviewers performed the article selection and data extraction (T.N, I.A.R, and Y·P·K). Any disagreements between the reviewers were settled through discussions with senior authors (J.R and L.H). The Cochrane risk of bias 2 (RoB 2) tool was used to evaluate RCTs’ qualities [17]. Bias due to confounding, due to participant selection, bias in intervention classification, due to deviations from intended interventions, due to missing data, bias in outcome measurement, and bias in the selection of reported results are all included in the instrument. Each of those domains was classified on two judgments, mainly some concerns or low concerns. Non-randomized studies were assessed with the Newcastle-Ottawa Scale (NOS) [18]. NOS score was considered good quality if they met the score equal to or more than six, while a score of five or less was considered poor quality. The quality of this systematic review was assessed using AMSTAR 2 criteria [19].

### 2.5 Outcomes

The primary outcome in this study was the UCF events following hypospadias repair. Secondary outcomes were other post-operative complications that increased the risk of UCF formation that including edema, infection, and wound dehiscence. All these four outcomes were ultimately analyzed in this study.

### 2.6 Statistical analysis

The fixed-effects model was selected if low heterogeneity was detected in between studies (I^2^ <50%; p-value ≥0.05). However, if the pooled analysis revealed high heterogeneity, the random-effects model was selected (I^2^ ≥ 50%; p-value<0.05). Because the extracted data is dichotomous, we presented the pooling analysis of our result in Odds Ratio (OR) with a 95% confidence interval (CI). If p-value <0.05, the result was regarded as significant. Statistical Software Review Manager 5.4 was used to analyze the studies (Cochrane Collaboration, Oxford, UK).

## 3 Results

### 3.1 Search result and baseline characteristics of study

**Fig. 1:**
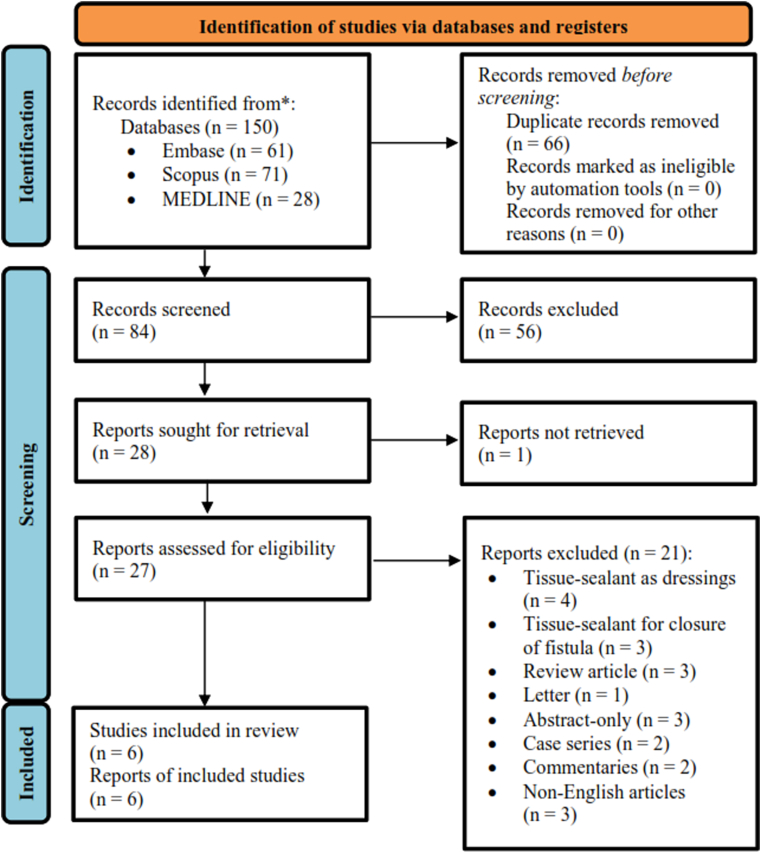
Study selection based on the PRISMA 2020 flowcharts.

The initial search turned up a total of 150 articles with 66 duplications, as displayed in Fig. 1. After the duplication removal process, we performed an initial screening for 84 articles by title, keyword, and abstract and we excluded 56 articles because not relevant. From the article sought for retrieval, we found one report not retrieved and found 27 articles assessed for eligibility. Finally, six eligible studies comprised of three RCTs and three non-randomized studies were enrolled for qualitative and quantitative synthesis. Hypospadias types in this study were proximal, distal, and midshaft. The techniques used in this hypospadias repair were Tubularized Incised Plate (TIP) technique, Thiersch-Duplay principle, Mathieu urethroplasty, MAGPI, double-face urethroplasty, and Duckett urethroplasty. Baseline characteristics of each included study were provided in Table 1.

**Table 1:** Baseline characteristics of included studies.

Author (year)	Design	Intervention	n	Age (months)	Hypospadias types	Technique	Follow-up
Ambriz- Gonzales et al. (2007)	Prospective cohort	Fibrin Glue	30	31.3 ± 24.1^a^	Proximal and Distal	Duckett, Thiersch-Duplay, TIP, Mathieu, ‘double face’ and MAGPI.	6 months
		Control	56	33.5 ± 19.2^a^			
Gopal et al. (2008)	RCT	Fibrin Glue	60	28.02 ± 9.56^a^	Proximal and Distal	Duckett urethroplasty	2 weeks, every 3 months for the first year, and every 6 months for the next 5 years
		Control	60	28.00 ± 10.3^a^			
Kocherov et al. (2013)	RCT	BioGlue	20	32.1 ± 11.0^a^	Proximal and Distal	TIP and *Mathieu*	3 months and 1 year
		Control	20	26.7 ± 7.72^a^			
Hosseinpour et al. (2019)	Case-control	Cryocalcium Glue	300	Not Reported	Distal	TIP	2 weeks and 3 months
		Control	100				
Nabil et al. (2019)	Prospective cohort	Fibrin glue	15	17 (10–32)^b^	Distal	TIP	4 weeks
		Control	15	17 (9–26)^b^			
Shenoy et al. (2021)	RCT	Fibrin glue	20	45.6	Proximal, distal and midshaft	TIP and Thiersch-Duplay	1 week, 1 month, 3 months, and 6 months
		Control	20	55.2			

a Data presented as mean ± SD; b: data presented as median (the minimum-maximum).

### 3.2 Risk of bias assessment among the study

**Fig. 2:**
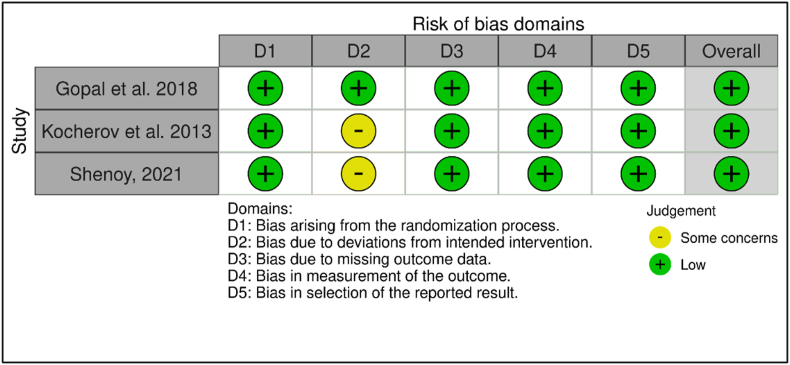
The Risk of Bias of included RCT using Cochrane RoB 2 tool.

The three listed RCTs in this study contained a low risk of bias, as shown in Fig. 2. Two research performed by Kocherov et al. [12] and Shenoy et al. [15] had some concerns in the bias domain due to deviation from intervention, as there was an inadequate description regarding the blinding process of both study subjects and the researcher. The quality assessment of non-randomized studies among inclusion demonstrated a moderate risk of bias. A study which was conducted by Hosseinpour et al. [13] had a low score due to the lack of clarity in the sample selection process for the possibility of objective evaluation. Therefore, the results showed a relatively high risk of bias, as shown in Table 2.

**Table 2:** The Risk of bias assessment of non-randomized studies using the Newcastle-Ottawa scale.

Author (Year)	Quality Score			
	Selection	Comparison	Exposure	Total
Ambriz Gonzalez et al. (2007)	***	*	**	6
Hosseinpour et al. (2019)	**	*	*	4
Nabil et al. (2019)	***	*	***	7

### 3.3 Outcome

#### 3.3.1 Urethrocutaneous fistula following hypospadias repair

**Fig. 3:**
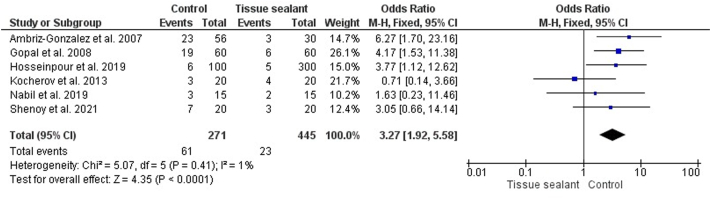
Forest plot of the urethrocutaneous fistula between the two groups.

A total of six matched studies were synthesized for pooled analysis. The total study population comprised 716 patients, categorized into control groups (n = 271) and patients receiving tissue sealant (n = 445). In our analysis, there was insignificant heterogeneity among the included studies (I^2^ = 1%, p = 0.41), as shown in Fig. 3. Therefore, we selected the fixed-effects model. According to pooled analysis, patients who underwent hypospadias repair without tissue sealant had a significantly higher risk of urethrocutaneous fistula compared to those who had tissue sealant (OR = 3.27; 95% CI 1.92–5.58; p = 0.0001).

#### 3.3.2 Edema following hypospadias repair

**Fig. 4:**
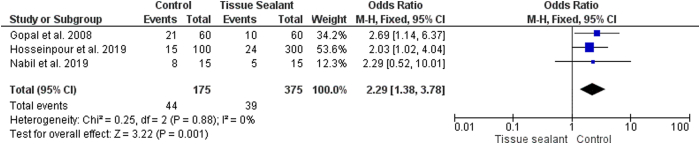
Forest plot of edema between the two groups.

In our analysis, we found three studies that reported complications of edema following hypospadias repair. Five hundred patients were categorized into the control group (n = 175) and the tissue sealant group (n = 375). Insignificant heterogeneity was found between the included studies (I^2^ = 0%, p = 0.88), as illustrated in Fig. 4. Accordingly, the fixed-effects model was chosen. Pooled analysis suggested that patients who underwent hypospadias repair without tissue sealant had a significantly higher risk of edema than those who had tissue sealant (OR = 2.29; 95% CI 1.38–3.78; p = 0.001).

#### 3.3.3 Infection following hypospadias repair

**Fig. 5:**
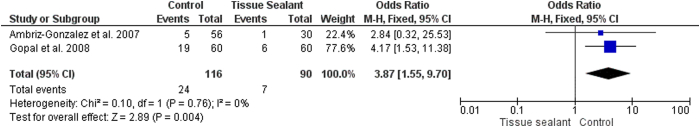
Forest plot of infection between the two groups.

There were two studies that reported complications of infection following hypospadias repair. A total of 206 patients were grouped into control (n = 116) and patients receiving tissue sealant (n = 90). There was insignificant heterogeneity among the included studies (I^2^ = 0%, p = 0.76), as illustrated in Fig. 5. Therefore, the fixed-effects model was selected. According to the pooled data analysis, patients who underwent hypospadias repair without tissue sealant had a significantly higher risk of infection than those who had tissue sealant (OR = 3.87; 95% CI 1.55–9.70; p = 0.004).

#### 3.3.4 Wound dehiscence following hypospadias repair

**Fig. 6:**
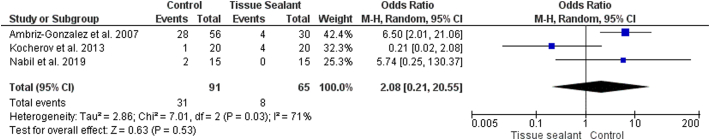
Forest plot of wound dehiscence between the two groups.

A total of 156 patients from three included studies were categorized as the control group (n = 91) and the tissue sealant group (n = 65). The heterogeneity among included studies was statistically significant (I^2^ = 71%, p = 0.03), as shown in Fig. 6. As a result, we selected the random-effects model for analysis. Pooled data analysis suggested that patients who underwent hypospadias repair without tissue sealant had a higher risk of wound dehiscence than those who had tissue sealant. However, this data was statistically insignificant (OR = 2.08; 95% CI 0.21–20.55; p = 0.53).

## 4 Discussion

Various hypospadias repair techniques and modifications have been developed to achieve good outcomes and reduce complications following hypospadias repair [10]. However, the complication rate following hypospadias repair remains high. The use of tissue sealant in hypospadias repair has gained interest in recent years. Tissue sealants are topical hemostatic agents and adhesive tissue approximations [20,21]. It comprises both natural and synthetic agents. Natural tissue sealant is made from polymers derived from protein and polysaccharides such as fibrin, collagen, gelatin, albumin, chitosan, and chondroitin, while synthetic tissue sealant is made from polycyanoacrylate, polyurethane, polyethylene glycol, and polyester [22,23]. As a hemostatic agent, several types of tissue sealant contain fibrinogen and thrombin. Tissue sealant facilitates blood clotting by mimicking the final step of the coagulation cascade and thereby accelerating the wound healing process [24,25]. Tissue sealants are also known for forming polymers that have strong adhesion to bind tissues are often applied as additives during urethroplasty [3,22].

The role of tissue sealant in preventing UCF following hypospadias repair has been investigated in several studies [3,[11], [12], [13], [14], [15]]. Our data showed that the occurrence of urethrocutaneous fistula was more common among controls, in comparison to patients receiving tissue sealant (OR 3.27; p < 0.0001). Based on the basic properties of tissue sealant, it can prevent extravasation of urine from the suture line which can break down the surgical anastomosis [3,[11], [12], [13], [14], [15],^26^]. The tissue sealant provides a watertight surgical anastomosis immediately after application during urethroplasty and the ability to resist hydrostatic pressure of urine at the surgical suture line [23,24]. Tissue sealant help to minimize and redistribute wound edges tension [21,24]. Tissue sealants also may improve urethral healing following hypospadias repair. A study by Ambriz-González et al. stated that fibrin-based tissue sealant may improve urethral healing following hypospadias repair by increasing the local proliferation of fibroblasts and reducing the local inflammatory response [11]. In addition, fibrin-based tissue sealants are known to facilitate cellular migration, enhance angiogenesis, and encourage the release of several growth factors that are important in the wound healing process [[27], [28]]. Nevertheless, the study conducted by Kocherov et al. stated no significant difference of urethrocutaneous fistula in the group receiving tissue sealant compared to those who did not [12]. This condition occurs because certain types of tissue sealants are known to trigger allergic reactions and tissue toxicity. This toxicity probably comes from a combination of tissue sealant ingredients, namely glutaraldehyde [12,29].

In this study, our analysis also demonstrated that tissue sealant could reduce the risk factors for postoperative complications that may contribute to the UCF event. Tissue sealant application significantly minimizes the risks of postoperative edema and infection. In hypospadias repair, the tissue was loose and fragile, susceptible to edema and infection, subsequently leading to UCF formation [30]. Tissue sealants are well known for their ability to create conjunction with sutures or tape to promote optimal wound integrity. It can effectively eliminate potential spaces and minimize the risks of edema and infection [3,21]. Tissue sealants are also known to have the ability to reduce serous fluid accumulation and seroma development which causes edema and infection that subsequently lead to wound dehiscence [[24], [31]]. In addition, our analysis also demonstrated that the group without tissue sealant application had a higher incidence of wound dehiscence than the group of patients receiving tissue sealant. However, the difference in wound dehiscence events was insignificant between the groups (OR 2.08; p = 0.53).

This review has several limitations. First, we could not perform a subgroup analysis of each hypospadias type and different surgical techniques due to the limited number of samples. Secondly, various factors might affect the results of this study, such as different characteristics of the patients, different types of tissue sealant, and different times of follow-up. Therefore, future randomized studies focusing on the effect of tissue sealant with larger sample sizes, similar patient characteristics, and treatment protocol while addressing these shortages are awaited.

## 5 Conclusion

Our data demonstrated the potential use of tissue sealant in hypospadias repair. Tissue sealant can reduce UCF events following hypospadias repair and other post-operative complications that can increase the risk of UCF development, like edema and infection. However, its impact on reducing wound dehiscence is not apparent. This agent could be used and have beneficial effects in hypospadias repair.

## Ethical approval

A systematic review does not require an ethical approval.The protocol for this review has been registered in PROSPERO data base (CRD42020198066) and Research Registry (reviewregistry1348).

## Sources of funding

The author received no financial support for the research.

## Author contribution

•Tomy Nurtamin (T.N) is involved in the concept and project design, materials, literature search,

data collection and/or processing, analysis and/or interpretation, writing the manuscript, and final

approval of the version to be submitted.

•Johan Renaldo (J.R) is involved in the supervision, resources, analysis and/or interpretation,

writing the manuscript, and and final approval of the version to be submitted.

•Yudhistira Pradnyan Kloping (Y.P.K) is involved in literature search, data collection and/or

processing, analysis and/or interpretation, and writing the manuscript.

•Ilham Akbar Rahman (I.A.R) is involved in literature search, data collection and/or processing,

analysis and/or interpretation, and writing the manuscript.

•Lukman Hakim (L.H) is involved in the concept and project design, supervision, resources,

materials, literature search, analysis and/or interpretation, writing the manuscript, and final approval of the version to be submitted.

## Registration of research studies

Name of the registry: PROSPERO international prospective register of systematic reviews and Research Registry.

Unique Identifying number or registration ID: CRD42020198066 and reviewregistry1348.

Hyperlink to your specific registration (must be publicly accessible and will be checked): https://www.researchregistry.com/browse-the-registry#registryofsystematicreviewsmeta-analyses/registryofsystematicreviewsmeta-analysesdetails/6263bc437ae706001ef3f805/ and https://www.researchregistry.com/browse-the-registry#registryofsystematicreviewsmeta-analyses/registryofsystematicreviewsmeta-analysesdetails/6263bc437ae706001ef3f805/

## Guarantor

Lukman Hakim, Department of Urology, Faculty of Medicine, Universitas Airlangga, Universitas Airlangga Teaching Hospital, Indonesia. Email: lukman-h@fk.unair.ac.id.

## Consent

This systematic review does not require an informed consent statement.

## Provenance and peer review

Not commissioned, externally peer-reviewed.

## Declaration of competing interest

The authors declare that there are no conflicts of interest.
